# Kaiso mediates transcription and RNA splicing in colorectal carcinoma: role of BRCA1 in the Kaiso enhanceosome

**DOI:** 10.1098/rsob.240329

**Published:** 2025-06-04

**Authors:** Weifeng Luo, Manish K. Tripathi, Qi Liu, Lei Chen, Robert W. Cowan, Juliet M. Daniel, Chi Yan, Ann Richmond, Albert B. Reynolds

**Affiliations:** ^1^ Department of Pharmacology, Vanderbilt University School of Medicine, Nashville, TN, USA; ^2^ Departments of Medicine and Oncology, University of Texas Health Rio Grande Valley, McAllen, TX, USA; ^3^ Department of Biostatistics, Vanderbilt University Medical Center, Nashville, TN, USA; ^4^ Department of Cell and Developmental Biology, Vanderbilt University, Nashville, TN, USA; ^5^ Department of Biology and Center of Discovery in Cancer Research, McMaster University, Hamilton, Ontario, Canada; ^6^ Pharmacology, Vanderbilt University School of Medicine, Nashville, TN, USA; ^7^ Pharmacology, Vanderbilt University, Nashville, TN, USA

**Keywords:** ZBTB33/Kaiso, BRCA1, transcription, colorectal cancer, RNA splicing

## Abstract

Kaiso (ZBTB33) is a transcription factor involved in mitotic clonal expansion and tumorigenesis in association with Adenomatous Polyposis Coli (APC) loss of heterozygosity. ENCODE data show strong overlap of the Kaiso promoter-binding site—encode-derived Kaiso-binding site (eKBS) and many other transcription factors, including BRCA1. Here we sought to determine whether BRCA1 is a component of the Kaiso enhanceosome that regulates gene transcription. Using proximal ligation assays, immunoprecipitation followed by mass spectrometry, luciferase assays and ChIP-seq experiments, we evaluated the association between BRCA1 and Kaiso. Kaiso nuclear extract immunoprecipitation experiments revealed that Kaiso associates strongly with genes involved in RNA splicing and processing. When Kaiso was not crosslinked to DNA, BRCA1 was not detected among Kaiso-binding proteins. However, overexpression of BRCA1 increased Kaiso-mediated gene transcription in luciferase assays in a Kaiso-dependent manner. Comparison of BRCA1 ChIP-seq and Kaiso ChIP-seq data from HCT116 cells revealed both BRCA1 and Kaiso commonly bind to the promoters of 379 genes. The most enriched term associated with these genes where BRCA1 and Kaiso bind their promoters is metabolism of RNA. Disease processes associated with these BRCA1/Kaiso gene promoters indicate that BRCA1 is functionally linked to a Kaiso-directed programme of RNAP2-mediated gene transcription and likely associated with colorectal cancer development and maintenance.

**Figure:**
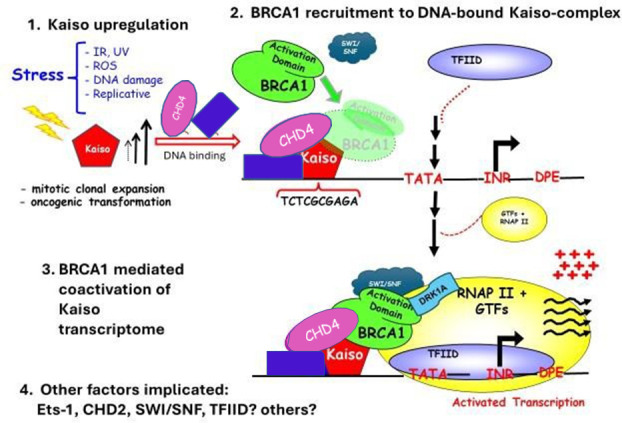

## 1. Introduction

Kaiso (aka ZBTB33) is a ZBTB family transcription factor (TF) identified originally as a specific binding partner of p120-catenin (p120) [1]. ZBTB family members are typically associated with important roles in differentiation, development and/or cancer. The localization of Kaiso in mammalian cells is largely nuclear, and it is sometimes localized in nuclear ‘dot-like structures’ associated with the centrosomes and midbody indicating a role for Kaiso in cell division [2]. The precise function of Kaiso is unknown, but it is agreed that it regulates transcription, though there is a major issue surrounding the identity of its DNA-binding site. The zinc-finger motifs of Kaiso bind methylated DNA [3,4] at the motif TCTCGCGAGA but only when both of the cytosines of the CGCG motif are methylated. However, there are reports that Kaiso also binds to unmethylated DNA at TCCTGCNA motif (Kaiso Binding Motif (KBS)) [1]. The methylated Kaiso binding consensus motif was recently validated by ENCODE using CHiPseq with Kaiso-specific mAbs generated in our laboratory. The binding motif identified by these antibodies is identical to ‘M8’ (TCTCGCGAGA), previously catalogued as an orphan motif corresponding to the eight most conserved DNA element in vertebrate genomes [5,6]. Hereafter, the encode-derived Kaiso-binding site will be referred to as the ‘eKBS’.

There are questions as to whether Kaiso binding to DNA represses transcription or activates transcription. Kaiso has been shown to suppress the transcription of methylated tumour suppressor genes in colorectal cancer [7]. This repression involves Kaiso recruitment of the histone deacetylase complex [8] to the DNA. Interestingly, it has been suggested that most methylated CGCG motifs are found wrapped around nucleosomes where the state of the chromatin is repressed [9]. However, the vast majority of eKBS motifs exist in very close proximity to the transcriptional start sites (TSSs) of approximately 3000 genes (hereafter, the Kaiso transcriptome), most of which are actively transcribed. Moreover, nuclear Kaiso levels are consistently high in colorectal tumour cells following APC Loss of Heterozygosity (APC LOH) and depletion of Kaiso prolongs survival and delays polyp formation in the Apc(Min/+) mouse model of intestinal cancer [10,11].

More recently, Kaiso has been described as a 5′methylcytosine reader that is often mutated in a pattern that is non-random producing missense variants in chromosomal haematopoiesis of indeterminate potential CHIP (clonal hematopoiesis of indeterminate potential) patients [12]. Beauchamp *et al.* showed that Kaiso (ZBTB33) interacts with splicing associated and mitochondrial proteins in hematopoietic cells. Mutations in Kaiso were described as drivers of clonal haematopoiesis and CHIP phenotypes [12]. In preadipocytes, Kaiso represses adipocyte differentiation and facilitates mitotic clonal expansion [13].

**Figure 1.**
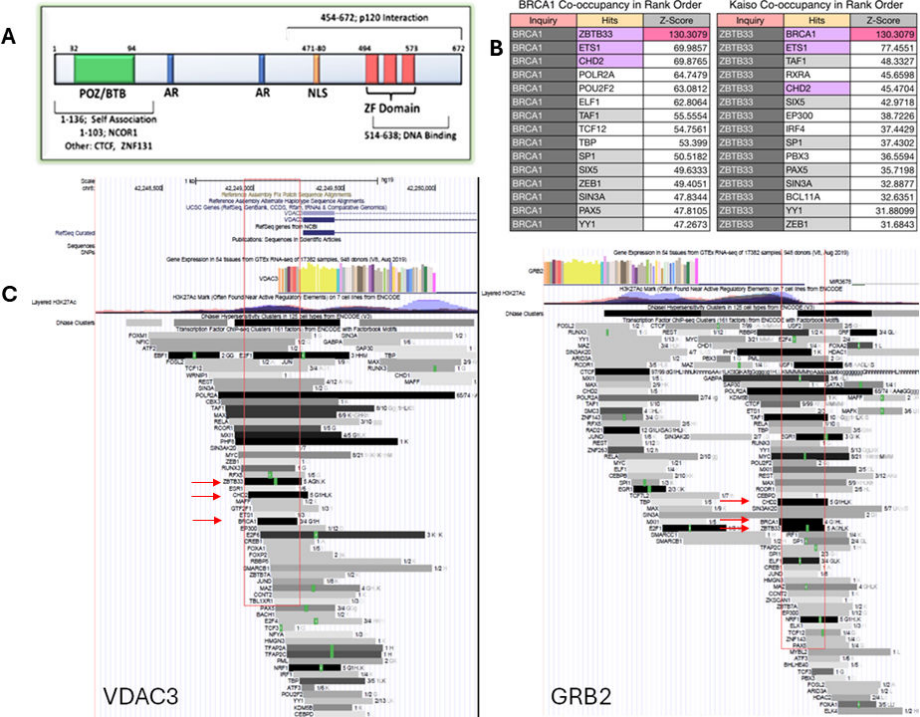
Visual inspection of VDA3 and GRB2 promoters (ENCODE ChIP-seq profile) (lower panels) and bioinformatic profiles (upper panels right) of top 15 ranked eKBS co-occupants for BRCA1 and ZBTB33/Kaiso across ENCODE data across multiple cancer cell lines. (A) Diagram of the Kaiso domains. (B) Co-occupants of BRCA1 and Kaiso/ZBTB33 are rank ordered by Z score. Purple fill mark top core hits. Light grey mark hits common to both. (C) The location of the eKBS in the VDA3 and GRB2 promoters is indicated by the ZBTB33/Kaiso peak; the BRCA1 and CHD2 peaks are highlighted with red arrows. eKBS, encode-derived Kaiso-binding site.

In addition to Kaiso, the eKBS is variably ‘co-occupied’ by other proteins, suggesting the potential assembly of one or more protein complexes that are directly or indirectly recruited to the eKBS by Kaiso. Of interest, Kaiso binding to the unmethylated Kaiso DNA-binding site can reduce the enhancer blocking activity of the CCCTC-binding factor (CTCF) insulator [14], but it is important to take into consideration that there are often differences between *in vitro* Kaiso binding studies and *in vivo* studies [9]. ENCODE data show a strong overlap between Kaiso-binding sites and the binding of the tumour suppressor BRCA1, and several RNA polymerase II (RNAP2)-associated factors, including POLR2A, and the core component of RNAP2 general transcription factor TFIID (among others; Figure 1), suggesting a cancer-relevant role in RNAP2-mediated transcription. Notably, BRCA1 does not have a sequence-specific DNA-binding domain of its own, suggesting that it might be indirectly tethered to the eKBS by Kaiso. DYRK1A, the dual-specificity tyrosine-phosphorylation-regulated kinase 1A also known as ‘the Downs syndrome kinase’, may also be a component of the putative ‘Kaiso enhanceosome’ in that it indirectly binds Kaiso and directly binds and phosphorylates p120, possibly modulating Kaiso transcriptional activity [15].

In this report using proximal ligation assays (PLA) and co-immunoprecipitation followed by mass spectrometry experiments, we sought to determine whether Kaiso forms a transcriptional regulatory complex with BRCA1 and identify Kaiso-associating proteins in the nucleus of human HCT116 colorectal cancer cells. While our PLA data indicated that Kaiso and BRCA1 associate, we did not detect BRCA1 in the Kaiso immunoprecipitation experiments from protein lysates where protein/DNA crosslinking was absent. We observed that many of the proteins that Kaiso binds are involved in splicing, suggesting an involvement for Kaiso in the splicing of heterogeneous RNA. We also compared the genes that bind Kaiso versus BRCA1 using the HCT116 ENCODE ChIPseq analysis for Kaiso and our ChIPseq analysis for BRCA1in HCT116 cells (which is not in the ENCODE database). Our analyses of ENCODE ChIPseq and BRCA1 ChIPseq data from HCT116 cells show that Kaiso binds to the 5′untranslated regions (UTRs) of 63 genes, exons of 82 genes, intergenic regions of 890 genes, introns of 929 genes, non-coding regions of 53 genes, the promoter of 2993 genes and TSS of 125 genes. In contrast, our BRCA1 ChIP-seq analysis of HCT116 cells shows binding to the promoter of 461 genes, exons of 26 genes, introns of 187 genes, and intergenic regions of 126 genes, 5′UTR of 8 genes and the TSS of 19 genes. Both BRCA1 and Kaiso bind to the promoters of 379 genes. Those genes transcriptionally regulated by Kaiso are frequently also co-regulated by TP53, BRCA1, MYC, FOXM1, MYCN, DYRK1A and BANP, along with a number of other transcription factors. In addition, there is high overlap with SRPK1, a key splicing regulator. Altogether, our data indicate that Kaiso appears to be associated with a complex multifactorial transcriptional enhancer that fine-tunes transcription and RNA splicing of thousands of genes that regulate metabolic and cellular responses to stimuli and biological processes, including mitotic clonal expansion and colorectal cancer.

## 2. Results

Based upon ENCODE data suggesting an association between BRCA1- and Kaiso-binding sites, we examined the possibility that BRCA1 may directly or indirectly be recruited to the eKBS. To examine the direct interaction of Kaiso with BRCA1, we used the β-catenin-mutated HCT116 line because it is one of the seven model cell lines (and the only colon line) characterized by ENCODE. Thus, high-quality HCT116 ChIPseq data on 24 transcription factors, including Kaiso (ZBTB33, Pol2-2, CEBPB, YY1, EZH2, SIN3A and ATF), is publicly available on the UCSC browser for integrative analysis (e.g. figure 1). We utilized three methods to examine physical interaction to evaluate whether BRCA1 associates with Kaiso: PLA (proximity ligation assay), co-immunoprecipitation, and ChIP analysis using Kaiso and BRCA antibodies

### 2.1. Visualization of Kaiso and BRCA1 interaction based upon proximal ligation assay

**Figure 2.**
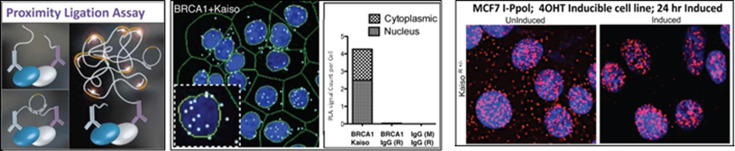
Kaiso and BRCA1 interact directly. Left three panels, BRCA1 and Kaiso interact specifically by PLA—the interaction takes place primarily but not exclusively in the nucleus. M, mouse, R, rabbit. Right panels: DNA damage causes striking translocation of Kaiso into the nucleus. Detection is by a single antibody variation of PLA. Each dot represents one molecule of Kaiso.

Our PLA data show that Kaiso and BRCA1 colocalize in the nucleus (figure 2). The PLA was performed with our Kaiso monoclonal antibody 6F (2 μg ml^−1^ of antibody) [16] and BRCA1 rabbit antibody (Cell Signaling cat. no. 9010, 0.25 μg ml^−1^). Moreover, using a tamoxifen-inducible MCF-7 breast cancer cell line that enables one to temporally control the generation of double stranded breaks, we show that DNA damage causes a striking translocation of Kaiso to the nucleus. One possible explanation is that BRCA1 recruits Kaiso to participate in DNA damage repair (DDR).

### 2.2. Identification of Kaiso-interacting proteins by immunoprecipitation followed by mass spectroscopy

**Figure 3.**
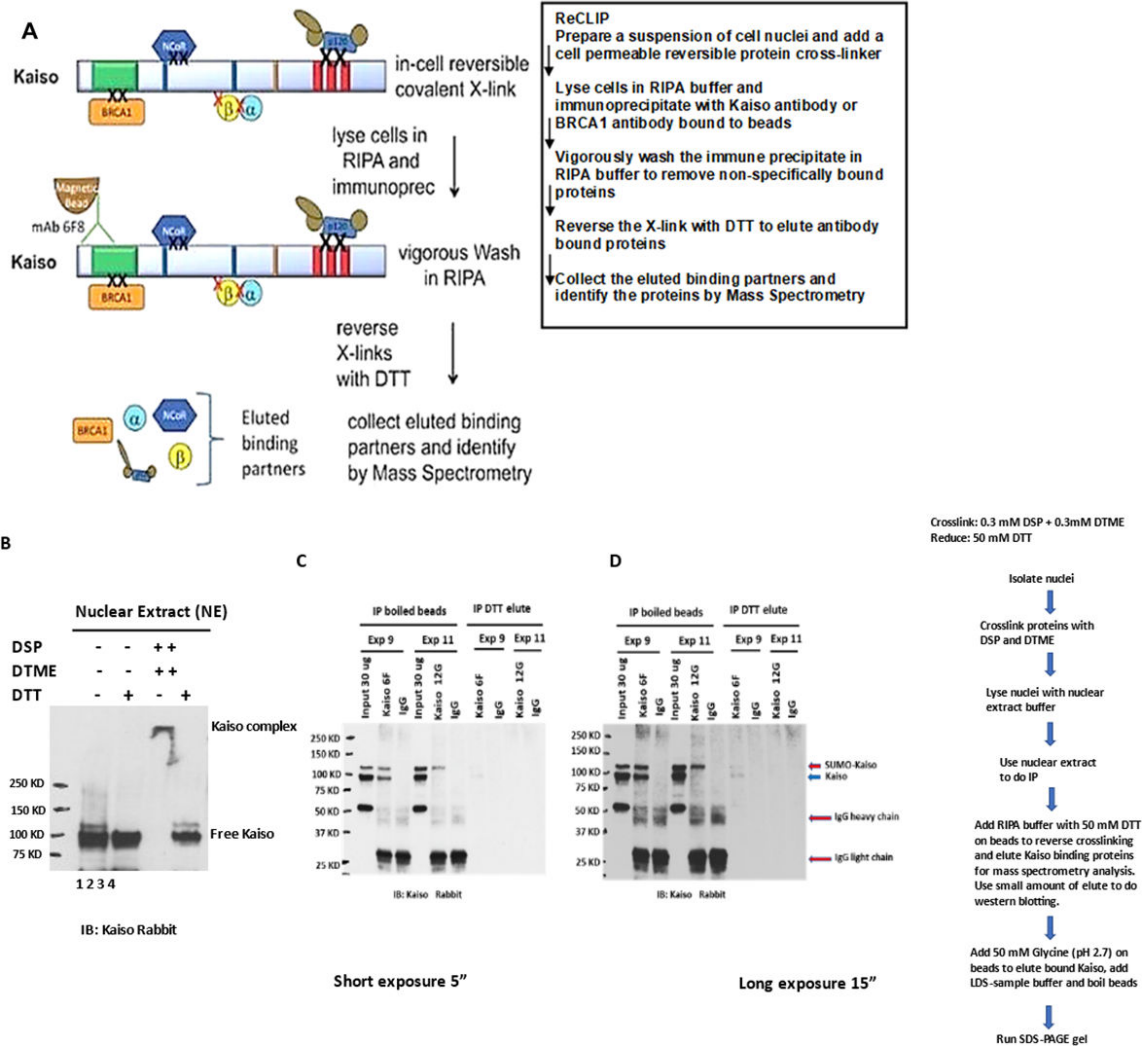
ReCLIP approach. (A) Key features of the method are shown: Crosslinkers (X) are (1) cell permeable and (2) cleavable, permitting ‘in cell’ X-linking to preserve protein–protein interactions as they occur in the cell. After vigorous washing, the sample is gently eluted with a reducing agent (DTT). The rest is discarded along with the majority of the potential background. Eluted protein binding partners are identified by mass spectrometry. (B) Western blot to check the crosslink efficiency of the Kaiso complex of crosslinked proteins. Nuclear extracts from DSP and DTME crosslinked cell nuclei (lanes 3 and 4) and non-crosslinked cell nuclei (lanes 1 and 2) were either treated with LDS-sample buffer without DTT (lanes 1 and 3) or LDS-sample buffer with DTT. In Lane 3, without DTT treatment, and subjected to western blot analysis using the polyclonal Kaiso antibody. Kaiso was seen in a large protein complex at the top of the gel. DTT reverses the crosslink and releases Kaiso from the complex as shown in Lane 4. (C) Immunoprecipitation of the Kaiso complex of proteins from HCT116 nuclear lysates. Protein/protein crosslinked nuclear extracts were used in immunoprecipitation experiments with either Kaiso 6F or 12 G mouse monoclonal antibodies. Normal mouse IgG was used as a control. RIPA buffer with 50 mM DTT was used to reverse crosslinking and elute Kaiso binding proteins for mass spectrometry analysis. A small amount of the elutes were subjected to western blotting with rabbit Kaiso antibody, labelled as IP DTT elute on the right of panel C. Bead bound Kaiso was boiled and eluted with 50 mM glycine (pH 2.7) and subjected to western blotting, labelled as IP boiled beads on the left of panel C. (D) Longer exposure of panel C.

To better approach the function of the Kaiso/BRCA1 complex, we conducted a proteomic interrogation using reversible crosslinked immunoprecipitation (ReCLIP; Figure 3A), a method developed recently in the Reynolds lab to both simplify and improve recovery of labile protein–protein interactions [17]. The method is now widely used [18]. The procedure is simple, extremely clean (remarkably high signal-to-noise ratio) and routinely captures weak or transient interactions that are inevitably lost by other methods. We first performed Kaiso immunoprecipitation experiments. In these experiments, Kaiso protein was first identified in non-crosslinked, or in Dithiobis-succinimidyl propionate (DSP) and Dithio-bismaleimidoethane (DTME) crosslinked HCT116 nuclear lysates with or without radioimmunoprecipitation assay (RIPA) buffer and DTT reduction using a Kaiso rabbit antibody for western blot. Without crosslinking and with or without DTT reduction (lanes 1 and 2 of figure 3B), Kaiso is predominantly a 100 KDa protein. With crosslinking but without reduction shows a high-molecular weight Kaiso complex near the top of the gel and with the addition of DTT the high-molecular complex is broken, and Kaiso is detected in 100 and 120 KDa forms (lanes 3 and 4). Next, Kaiso protein complexes were isolated by ReCLIP from HCT116 cells (figure 3C). Here, the proteins in the nuclear lysates were first reversibly crosslinked and then subjected to immunoprecipitation with Protein A/G beads bound to either the Kaiso 6F or 12G monoclonal antibodies that were previously developed and characterized in the Reynolds lab [1]. Very little Kaiso protein was eluted by DTT because the Kaiso binding to 6F and 12G antibody is not di-sulfide dependent, and the affinity of Kaiso for the antibody bound beads is stronger than the linkage of Kaiso to its associating proteins. We see faint bands that represent both the 120 KDa band and the 100KDa band were brought down by the 6F antibody but with the 12G antibody, the 120 KDa form of Kaiso is the predominant form detected (figure 3C; DTT eluant experiment 1 with 6F, lanes 7−9) and experiment 2 with 12G, Lanes 10−12. The additional approximately 120 KDa Kaiso band likely represents the SUMOylated form of Kaiso [19]. ReCLIP eluates from HCT 116 cells were prepared and analysed by mass spectroscopy (MS) in the Vanderbilt Proteomics Core. To control for nonspecific binding, we used isotype-matched monoclonal epitope tag antibodies. It is worth noting that ReCLIP is ideal for bringing down intact complexes because the interacting subunits are covalently crosslinked prior to precipitation and washing.

After DTT elution, the Kaiso-associating proteins bound to antibody beads were eluted by boiling and subjected to western blot using the Kaiso rabbit polyclonal antibody (lanes 1−6 of figure 3C and D). The rabbit polyclonal Kaiso antibody also detects an approximately 52 Kd non-specific band in the lysates. Boiling increased the elution of Kaiso but also resulted in elution of the IgG heavy and light chains.

Results from the top hits identified with the 6F and 12G antibody immunoprecipitates as well as IgG controls are shown in electronic supplementary material, table S1A (all hits). As expected from the initial pull-down experiments, the Kaiso 6F antibody IP pulled down larger quantities of peptides from each identified protein. Of interest, the 12G antibody probably recognizes the SUMOylated form of Kaiso, which is thought to play a role in Kaiso’s function as a transcriptional activator [20]. We performed gene-enrichment analysis for molecular function and biological processes linked to Kaiso-associating proteins (figure 7). In this analysis, functional pathway enrichment of over-representation analysis was performed on the top 85 Kaiso associating proteins based upon immunoprecipitation and mass spectroscopy analysis using the PANTHER Go-SLIM package (v. 19).The 85 Kaiso-associating proteins selected for pathway analysis represent the genes with a high ratio of peptides captured by the 6F Kaiso antibody immunoprecipitation showing a minimum of 2.5 fold more peptides as compared with the control IgG immunoprecipitation.

Interestingly, Metascape analysis [20] of the pathways associated with Kaiso binding proteins as well as analysis of the functional and biological pathways associated with Kaiso binding proteins revealed that the major pathway for Kaiso associated proteins is RNA splicing, followed by spliceosome, then regulation of RNA splicing, HEXIM-DNA-PK- paraspeckle components-ribonucleoprotein complex, mRNA 3’-end processing, chromatin remodelling, heterochromatin organization and DNA transcription (figure 7B).

**Figure 4.**
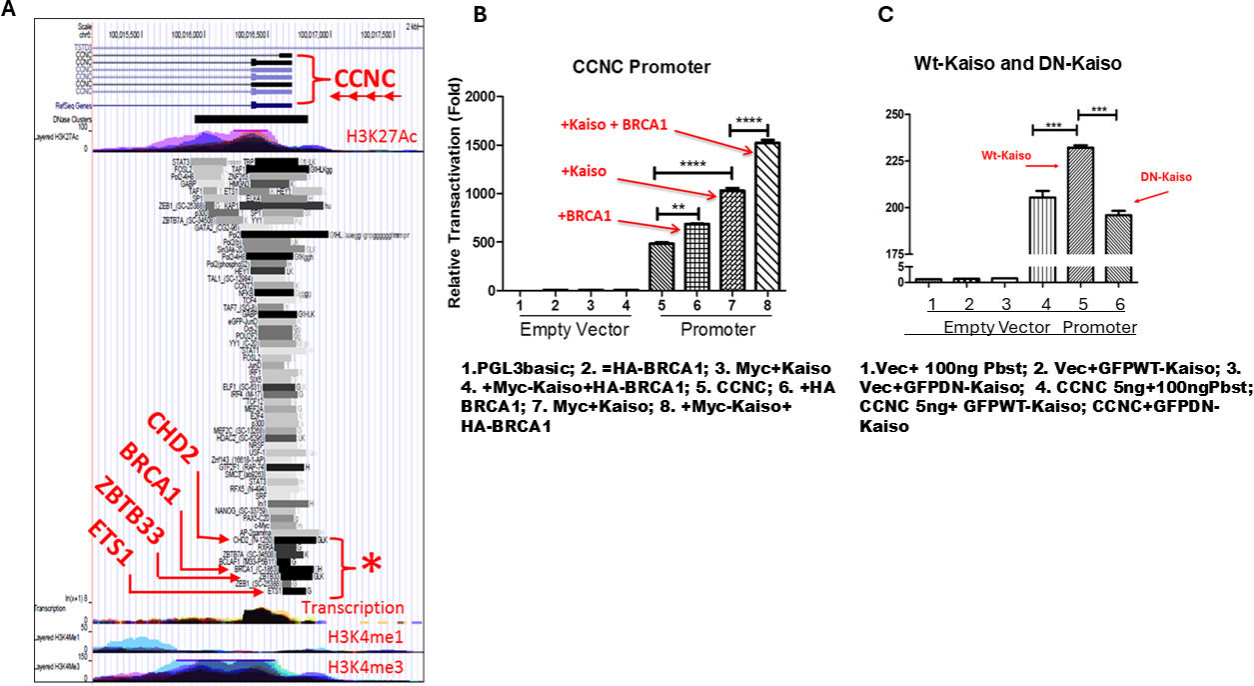
(A) Encode data showing how CHD2, BRCA1, ZBT33 and ETS binding sites overlap on the CCNC promoter. (B) BRCA1 transactivates Kaiso as eKBS sites. The CCNC promoter was subcloned into the ‘empty’ vector PGL3basic and assayed for *in vitro* transcriptional activity after transient transfection of the empty vector (lanes 1−4) or the same vector expressing the constructs indicated across the bottom. (C) As in (B), but the experimental constructs compare the effects of endogenous Kaiso (lane 4), to transiently overexpressed Kaiso (lane 5) and a dominant negative (DN) Kaiso (lacking N-terminal BTB domain; lane 6). BRCA1 elevates transcription compared with Kaiso overexpression alone by 30%. DN-Kaiso suppresses transcription relative to endogenous Kaiso alone.

It is puzzling that Kaiso appears to activate transcription at the eKBS and repress transcription at the KBS. To confront this issue directly, we generated defined natural CCNC promoter-luciferase reporter construct (CCNC is a gene known to be regulated by Kaiso-mediated transcription; figure 4A). Similar constructs have been tested previously for DYRK1A activity [15] and Kaiso [21–23], respectively, but the experiments differ markedly in many respects including construct design and cell types tested. We examined the effects of expression of HA-BRCA1, Myc-Kaiso, GFP-Kaiso, GFP DN Kaiso, HA-BRCA1 + Myc-Kaiso or empty PGL3 vector in HEK293 cells on the CCNC-luciferase reporter construct that includes an eKBS element [24]. For the CCNC reporter construct, we observed a small but significant increase in luciferase activity with BRCA1 overexpression, an over twofold increase in luciferase activity when Kaiso was overexpressed, and an approximately threefold increase in luciferase activity in cells expressing Kaiso + BRCA1 (figure 4C). We also observed that when a DN-Kaiso was expressed, there was a reduction in luciferase reporter activity (figure 4C). We observed that Kaiso enhanced the promoter luciferase activity of four additional genes with eKBS containing promoters, HNRNPK, RPS9, RPS11 and RPS19 (electronic supplementary material, figure S4). We observed similar increased luciferase activity when BRCA1 was expressed with Kaiso in experiments with RPS9 and RPS19, but not RPS11, CDK8, CDC5L and HNRNPK luciferase reporters, though in these gene promoters BRCA1 and Kaiso both enhanced luciferase activity of the respective genes, but the combination was not better than BRCA1 or Kaiso alone(electronic supplementary material, figure S4).These data suggest that BRCA1 can enhance eKBS-mediated transcription for several genes. The results in figure 4 show that (i) Kaiso can promote transcriptional activation from a natural eKBS-promoter and moreover, that (ii) BRCA1 can transactivate this Kaiso anchored activity for some but not all genes with an eKBS in their promoters. Notably, BRCA1 is well known to contain an autonomous C-terminal *trans*-modulation domain capable of strongly activating or repressing (depending on context) a heterologous promoter [25–27]. These data link Kaiso to BRCA1, and BRCA1 to a previously unappreciated Kaiso transcriptome enriched for genes involved in activities such as transcription, translation, DDR and cell cycle control. We suggest, therefore, that BRCA1 and other proteins likely co-occupy the eKBS along with Kaiso and this complex likely functions in the RNAP2-mediated transcription of eKBS target genes. This concept was examined further by ChIP-seq experiments.

### 2.3. ChIPseq analysis of the transcription factors associating with the encode-derived Kaiso-binding site in HCT116 cells

**Figure 5.**
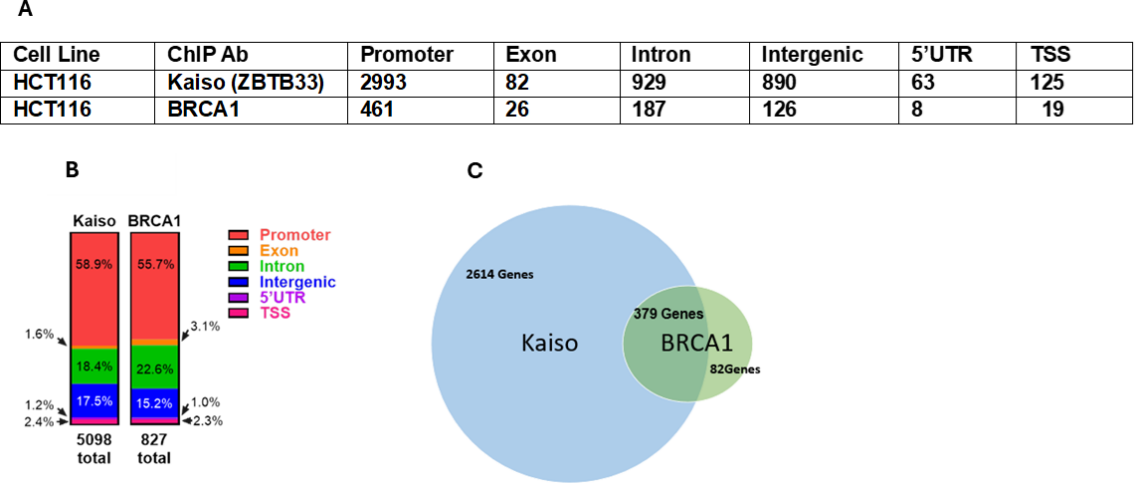
ChIP-seq analysis of Kaiso and BRCA1 DNA-binding sites. (A) Number of gene promoters, exons, introns or intergenic regions bound by Kaiso or BRCA1 antibody in HCT116 cells. (B) Distribution of Kaiso and BRCA1 antibody binding to various regions of the genes. (C) sVenn diagram visualizing the gene promoters with or without overlap of BRCA1 and Kaiso antibody binding.

**Figure 6.**
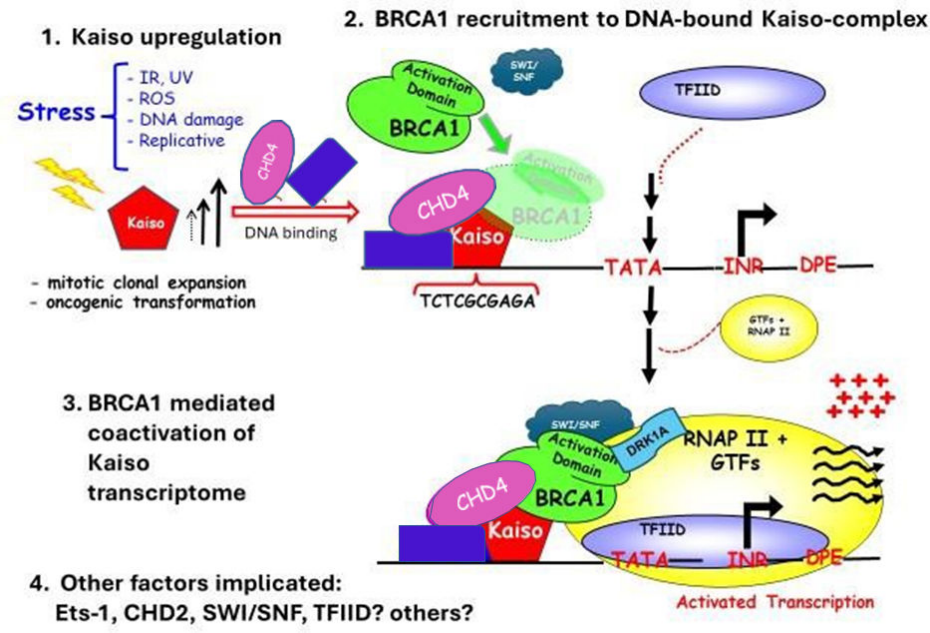
Schematic showing hypothetical interactions of eKBS co-occupants in proximal promoter transcriptional elongation. Components of the complex are anchored to DNA via Kaiso, which binds directly to the TCTCGCGAGA motif (aka the eKBS) near the transcriptional start site (TSS). BRCA1 is recruited to the CHD4/Kaiso complex, RNA Polymerase II+GTFs bind to BRCA1 at the TATA box of a promoter, and transcription is activated with the help of INRs.

ChIP-seq analysis was performed on HCT116 cells expressing or not expressing Flag-Kaiso. Cell nuclear DNA and proteins were crosslinked with formaldehyde and protein/protein interactions were captured with DGS crosslinking. Immunoprecipitations of crosslinked DNA/proteins were performed with BRCA1 antibody (Cell Signaling, cat. no. 9010) or IgG control antibody of the same class. Numerous binding events were captured in promoter, exon, intron and intergenic regions. Data from our ChIPseq analysis of BRCA1 binding were compared with the ENCODE Kaiso binding, combining the data from multiple experiments for Kaiso ChIP-seq. Figure 5A shows the number of genes with antibody binding sites for Kaiso and BRCA1 antibodies in the promoter, exon, intron, intergenic, 5′UTR and TSS. Figure 5B shows an analysis of the data in figure 6A. Figure 5C shows the genes with both Kaiso and BRCA1 binding to their promoters .

Electronic supplementary material, table S2, shows the overlap between BRCA1 (from our ChIP-seq data) and Kaiso-binding sites (based on ENCODE analysis) in HCT116 cells (columns 1 and 2). Gene enrichment analysis of Kaiso regulated genes (identified by ChIPseq analysis) based on molecular function, biological process and pathways associated with Kaiso regulated genes, revealed a strong association of the Kaiso complex and RNA processing (figure 4A,B). Additionally, Metascape analysis of the human disease-associated genes and variants identified in the ChIP data revealed a striking relationship to multiple polyps, Lynch syndrome, Marinesco–Sjogren syndrome, mucinous adenocarcinoma and hereditary nonpolyposis colorectal cancer, implying a strong role for Kaiso regulation of genes associated with colorectal cancer development, leukaemia, hereditary diffuse gastric cancer, pancreatic cancer, cervical cancer, respiratory distress syndrome and breast cancer [28] (figure 5). Moreover, analysis of enriched terms across the list of genes for which Kaiso binds showed estrogen receptors (ESR)-mediated signalling, ncRNA metabolic process, multicellular organismal-level homeostasis, MAPK family signalling cascades, positive regulation of cell projection organization and diseases of signal transduction by growth factor receptors and second messengers figure 8 .

When the specific binding sites that were common between the BRCA1 and Kaiso monoclonal antibody and the BRCA1 and Flag-Kaiso antibody were evaluated, several key common sites were observed (electronic supplementary material, table S2). Whether these interactions result in positive or negative regulation of transcription of these genes remains to be determined, though we do observe that when BRCA1 is over-expressed, Kaiso-mediated transcription in HCT116 cells was enhanced (figure 4B).

When we analysed the gene sets that bind both Kaiso and BRCA1 to their promoters, a number of interesting relationships were revealed. Of note, metabolism of RNA stood out as the most significant term associated with genes that bind both BRCA1 and Kaiso to their promoters. Other highly relevant terms are associated with nonsense-mediated decay, chromosome organization and DNA metabolic processing (figure 9). Based upon the mass spectroscopy analysis of Kaiso-associating nuclear proteins where RNA processing and RNA splicing were the most frequently associated terms, it is quite interesting that Kaiso and BRCA1 are highly involved in the regulation of genes involved in RNA metabolism. Interestingly a recent report shows that mutations in Kaiso result in increased genome wide intron retention [12]*.* Moreover, given the role of Kaiso in promoting mitotic clonal expansion, the observation that cell cycle was shown to be a highly significant enrichment term for genes co-regulated by Kaiso and BRCA1. Moreover, the identification of DYRK1A as the third most enriched transcription factor targets associated with the Kaiso/BRCA1 regulated genes is quite intriguing, given the known association of p120 with DYRK1A and Kaiso and the data showing that DYRK1A ChIPs to the eKBS [20] (figure 8).

Transcriptional enhancers are known to include the binding and interaction of a number of transcription factors with the promoters of genes. ChIP-seq data show here that there is an association of BRCA1 and Kaiso with Pol2 and additional use of sentence-based text mining using the TRRUST reference database of human transcriptional regulatory interactions available in Metascape, we see that in addition to BRCA1, TP53, MYC, MYCN, FOXM1, AR, ESR1, STAT3 and numerous other transcription factors are potentially involved in the enhancer structures that involve Kaiso-mediated gene transcription [21] (figure 9C).

Of note, when we analysed the ENCODE data of gene promoters in MCF7 breast cancer cells that also bind BRCA2 (BRCA1 antibody is not reported in the ENCODE database) and Kaiso, we identified a generous list of genes, though fewer than co-bind gene promoters in HCT116 cells (electronic supplementary material, table S3A). Only six gene promoters were identified in both MCF7 and HCT116 cells as binding both BRCA1 (or BRCA2 in MCF7 cells) and Kaiso (ADNP, RBM39, RNF167, SKA2, TRIB1, WBPIL) (electronic supplementary material, table S3B) . Interestingly, there was little commonality between Kaiso/BRCA1 GO enrichment terms (electronic supplementary material, table S3C) or promoter binding genes in HCT116 cells and Kaiso/BRCA2 promoter binding in MCF7 cells as to enrichment of transcription factor targets (electronic supplementary material, table S3D), but there was some overlap in the summary of enrichment analysis in transcriptional regulatory relationships (TRRUST) showing commonality for ATF4, BRCA1, STAT3 and TP53 (electronic supplementary material, table S3E). Analysis of the genes that bind Kaiso in both MCF7 cells is shown in electronic supplementary material, table S3 F, G and H, and Metascape analysis of genes that bind Kaiso in HCT116 cells are shown in electronic supplementary material, table S3 I and S3J*.* Thus, Kaiso gene regulation appears to differ significantly between cell types.

## 3. Discussion

Identified originally as an interaction partner of p120-catenin, Kaiso (aka ZBTB33) belongs to a superfamily of BTB/POZ and Zinc Finger (ZF) domain-containing TFs (hereafter referred to as the ZBTB family) most of which are involved in adult tissue differentiation and cancer [22,29,30]. For well over a decade, the mouse small intestine has been used as a model to better understand the relationship between stem-progenitor cell differentiation and intestinal tumorigenesis. In most tissues, self-renewing adult stem cells give rise to progenitors, which in turn undergo several uninterrupted rounds of mitotic clonal expansion (MCE) to meet a steady-state requirement for new cells. Owing to enormous cell turnover, this early phase of the cell differentiation process is grossly accentuated in the crypts of the small intestine, giving rise to a prominent ‘transit amplification’ (TA) compartment. We demonstrated previously that strongly upregulated Kaiso expression coincides precisely with progenitor MCE as the cells migrate up through the TA compartment [31]. Kaiso has been identified in the mitotic spindle in mitotic entry [32]. Kaiso expression is then abruptly extinguished as the cells leave the intestinal crypt to emerge onto the villus as mature, growth arrested and fully (terminally) differentiated cells. In contrast, Kaiso expression is maintained indefinitely at very high levels in nascent GI tumours following loss of *Apc* [31].

Highly analogous behaviour is exhibited by ZBTB family members BCL6 (aka ZBTB27) [33] and the *Drosophila* Abrupt protein (probably ZBTB14) in blood cancers. BCL6 is a master regulator of the germinal centre reaction, which includes B-cell somatic hyper mutation to increase antibody affinity, class switch recombination and proliferation (i.e. MCE) [34,35]. BCL6 expression maintains germinal centre B-cell identity by repressing target genes that would otherwise interrupt affinity maturation (e.g. p53) and genes that would promote premature differentiation into plasma cells (e.g. PRDM1), while supporting the MCE of B-cell progenitors. However, for the terminal phase of this process (including growth arrest), BCL6 must be turned off. The failure to do so blocks terminal differentiation and is responsible for 40% of diffuse large B-cell lymphomas [36,37]. In both GI and blood cell systems, these ZBTB proteins function as master regulators over most of the differentiation process. They are then silenced to enable ‘terminal’ differentiation and growth arrest. It is the mechanistic coupling to growth arrest that distinguishes ‘terminal’ differentiation from earlier events in the process that go on simultaneously through multiple cell divisions without triggering growth arrest. Still another Kaiso-like ZBTB family member is ZBTB38, which, like Kaiso, binds methylated DNA at CpG islands and/or shores. ZBTB38 binding is predominately localized at transcriptionally active sites, while Kaiso binds at both active and inactive DNA-binding sites [38].

A long-standing research paradigm for Kaiso is that it functions predominantly as a transcriptional repressor, as do most ZBTB transcription factors. Nonetheless, our experiments based on our ChIPseq, and immunoprecipitation experiments indicate that Kaiso may be equally involved in RNA processing, based upon the fact that it frequently binds introns and inter-intronic regions of the DNA (figure 5)**.** A potential explanation comes from our discovery that BRCA1 appears to mostly transactivate Kaiso at a novel battery of eKBS-containing promoters. We postulate that the Kaiso transcriptome is strongly upregulated during MCE, abruptly downregulated at terminal differentiation and maintained aberrantly at very high levels upon nascent adenoma formation.

Individual components of the Kaiso regulatory complex are expected to contribute distinct functions (e.g. DRK1A may be selectively recruited to the Kaiso complex to drive gene-specific transcriptional elongation) [15]. Though we did not detect direct binding of Kaiso and CHD2 in immunoprecipitation/mass spectrometry experiments, we did observe its binding to CHD4, TAF15, TOP1, SMARCA5, SAFB2 and SAFB (electronic supplementary material, table S1). TAF15 is a component of the TFIID complex, TOP1 is topoisomerase 1, SAFB2 represses ERα and SAFB assembles transcriptional complexes. Of course, the conformation of Kaiso when it is bound to DNA may result in different protein/protein associations than are observed in the nuclear complexes not bound to DNA. The Kaiso-regulated transcriptional programme likely shuts down in normal intestinal epithelial cells when the Wnt gradient is sufficiently weakened and can no longer shut down Kaiso [10]. As an obvious corollary to our hypothesis, we suggest that failure to shut down Kaiso is the defining event with respect to the transition to tumorigenesis. In this report, we propose a gene-specific core complex that is built around Kaiso and selectively anchored to a battery of promoters identified by the presence of an eKBS which may form a putative ‘Kaiso enhanceosome’ (figure 6).

We suggest further that functions mediated by this protein complex will not be limited to transcription but rather will reflect multiple tightly coordinated activities, including RNA splicing (yellow highlighted genes in electronic supplementary material, table S1), that together execute the differentiation programme. With regard to regulation of RNA splicing, Kaiso associates with numerous splicing factors, both DNA and RNA splicing (electronic supplementary material, table S1). Kaiso is seen in dot-like structures in the nucleus and also co-associates with all of the core proteins from nuclear paraspeckles (NONO, SFPQ and PSPC1) as well as other proteins directly involved in paraspeckles (MATR3, RBMX, the HNRNP genes, SRSF9, ADAR, TAF15, etc). Paraspeckles are also at the site of RNA splicing, another key function of Kaiso. Finally, we believe that Kaiso is backed up by an at least partially redundant family member, possibly ZBTB14, accounting for the roughly 50% attenuation of APC-mediated tumorigenesis upon removal of Kaiso alone; in particular, the CML example illustrates a potent synthetic lethal interaction between BCL6 and the activated Abl tyrosine kinase [35].

**Figure 7.**
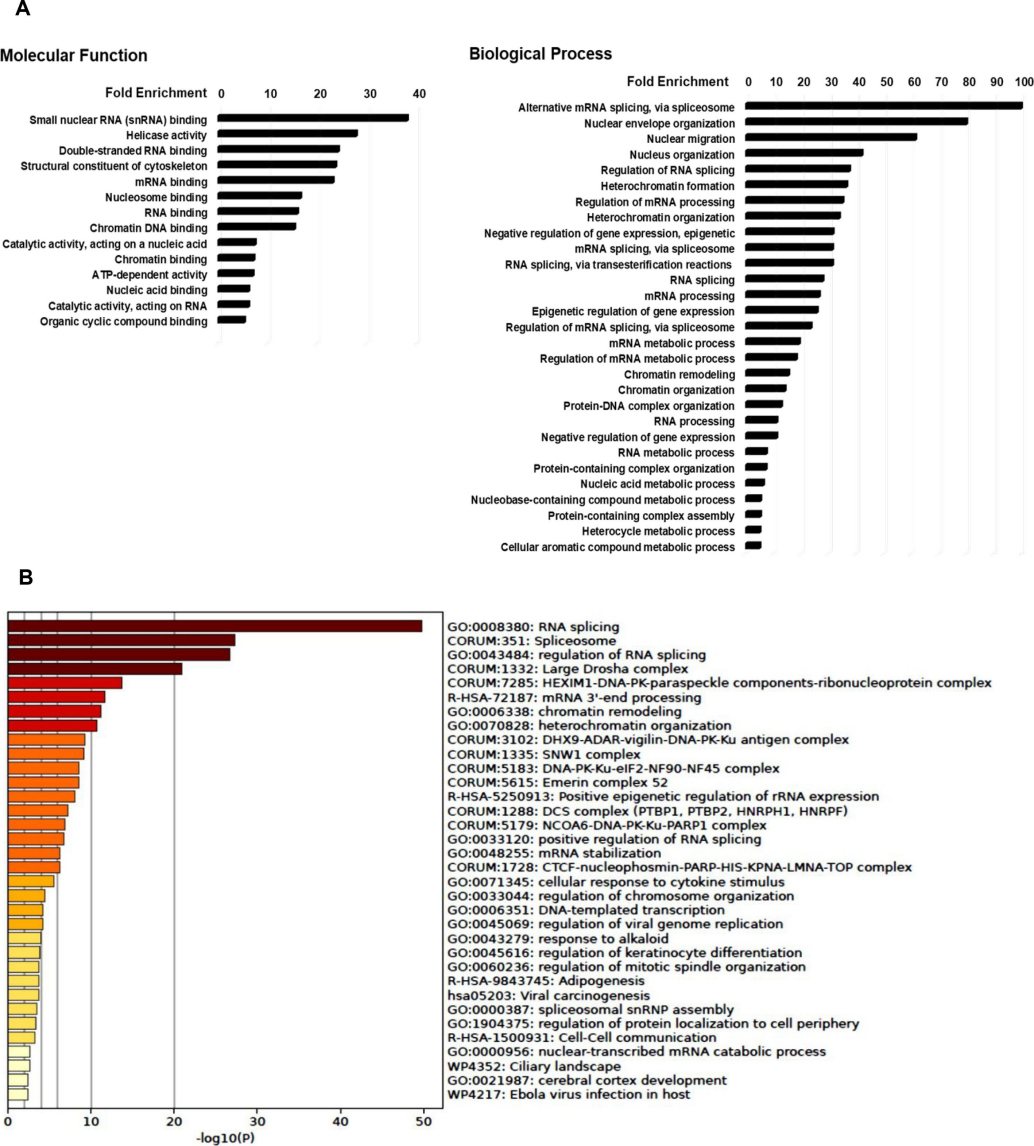
(A) Gene enrichment analysis for molecular function and biological processes linked to Kaiso associating proteins. (B) Pathways associated with Kaiso associating proteins identified by metascape analysis

The p120/Kaiso interaction is in many ways reminiscent of the β-catenin/TCF4 partnership and has long been viewed as a potential p120 pathway into the nucleus [39]. While BRCA1 has been linked previously to transcriptional pausing [40], DYRK1A is now implicated in the release phase of the Pol II elongation process, although its activity in this regard is apparently restricted to eKBS-delineated genes. Considered separately, the p120/Kaiso- and p120/DYRK1A-interactions have heretofore been viewed as independent activities. Together with our data, however, the surprising dependence of DYRK1A activity on the eKBS provides strong evidence that these interactions are functionally linked to a Kaiso-directed programme of RNAP2-mediated gene transcription and likely associated with the tumorigenic effects of APC-LOH. One possibility is that DYRK1A-mediated phosphorylation of p120 is one of the steps involved in the activation of Kaiso-mediated transcription figure 7.

**Figure 8.**
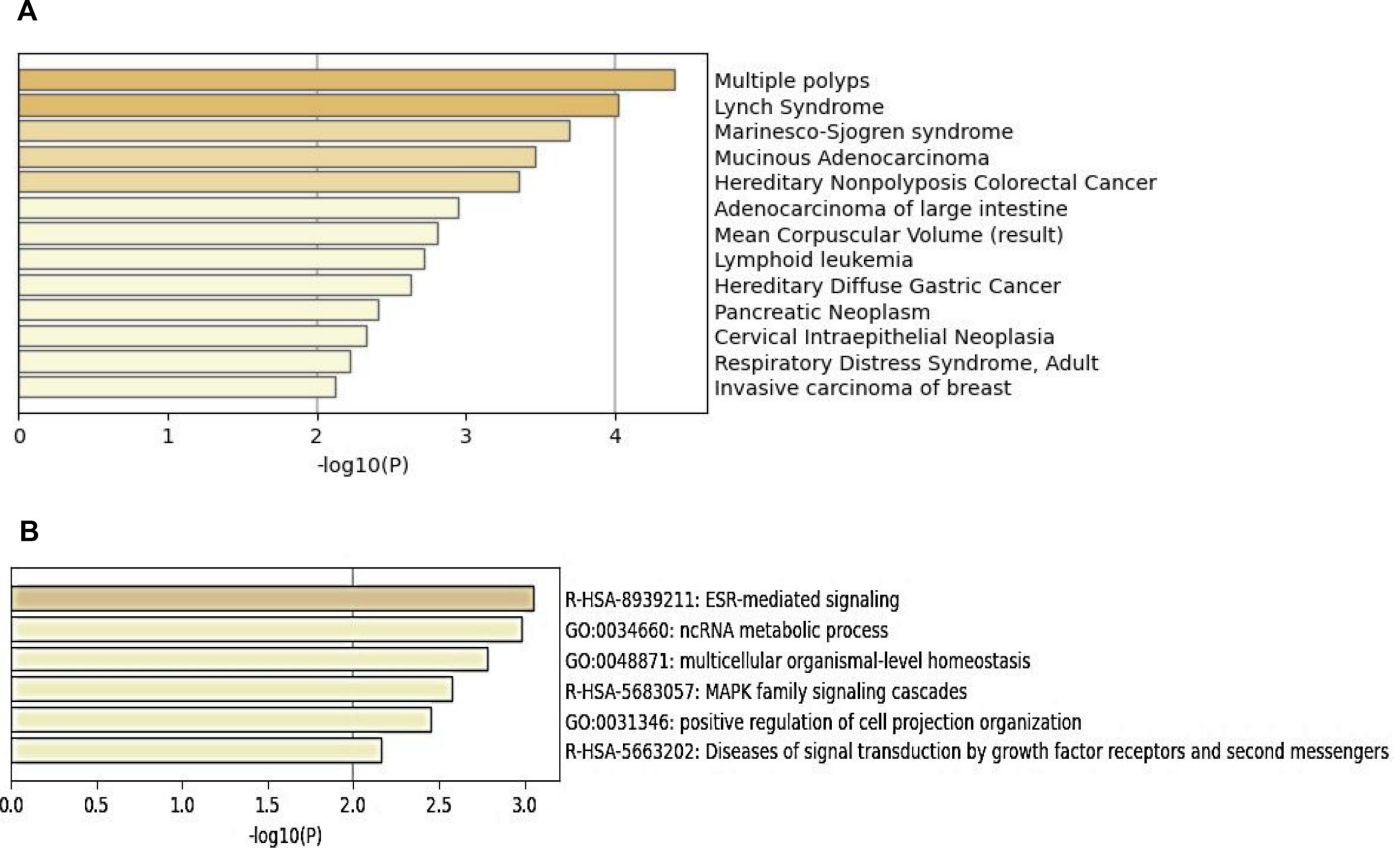
(A) Summary of enrichment analysis of HCT116 cell Kaiso binding genes in DisGeNET [16]. (B) Bar graph of enriched terms across input gene lists for Kaiso binding genes in HCT116 cells, coloured based on *p-*values.

Finally, our data show that there are clearly cell line differences in the genes that are transcriptionally regulated by both BRCA and Kaiso, but there are some common themes that emerge as to the biological functions that are controlled by the BRCA1/Kaiso enhanceosome complex. However, a limitation of our data from the MCF-7 ChIPseq analysis is that only BRCA2 data were available in the ENCODE database, and it is quite possible that BRCA1 and BRCA2 could influence epigenetic landscapes in divergent ways. Moreover, variations in enhancer activity of BRCA1 or BRCA2 might create differences in gene transcription that could not be attributed solely to Kaiso’s regulatory activity figure 8.

**Figure 9.**
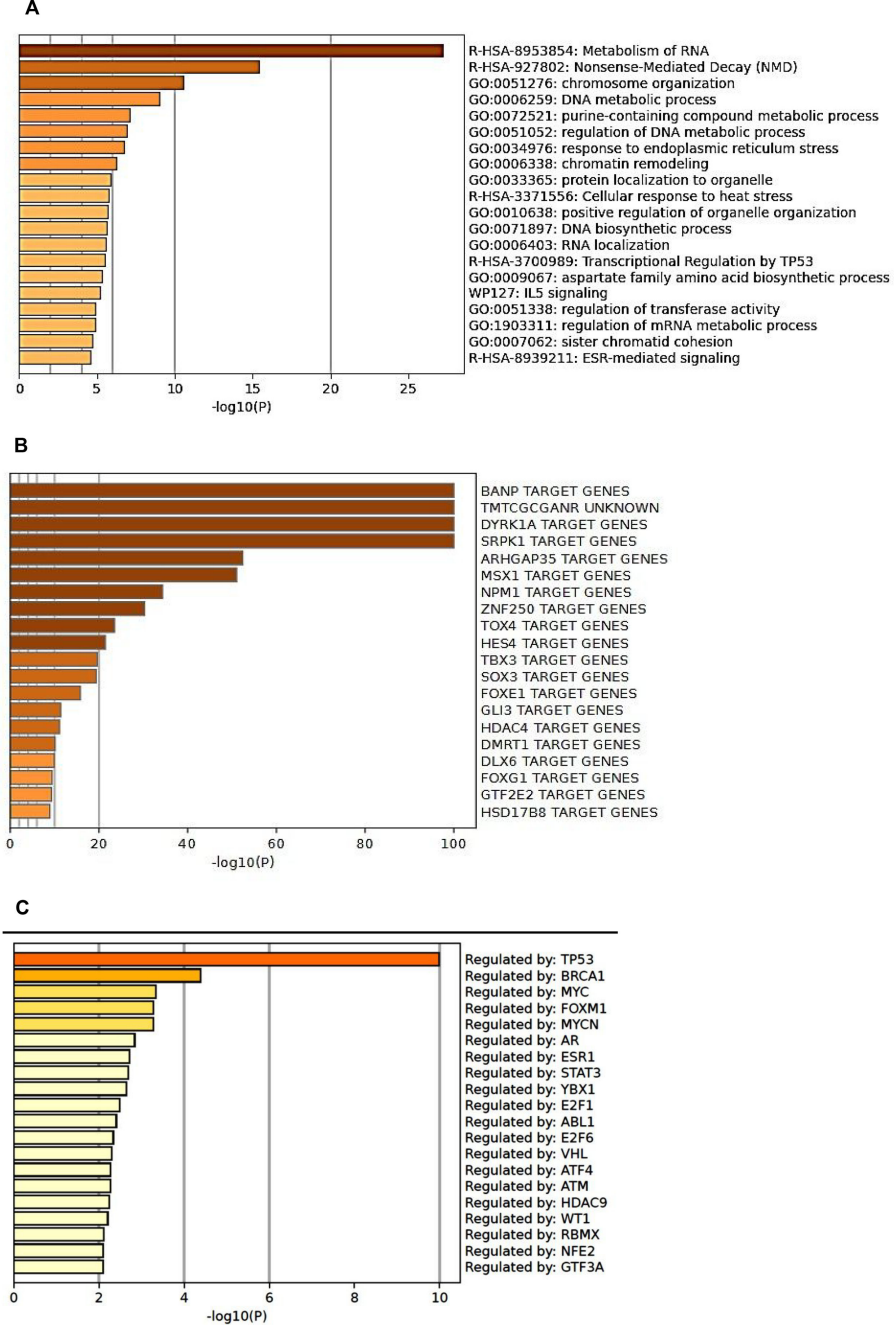
(A) Bar graph of enriched terms across input gene lists that bind both BRCA1 and Kaiso in HCT116 cells, coloured by *p*-value. (B) Summary of enrichment analysis in transcription factor targets from analysis of genes binding both BRCA1 and Kaiso in HCT116 cells. (C) Summary of enrichment analysis in transcriptional regulatory relationships unraveled by sentence-based text mining (TRRUST) analysis of genes binding both BRCA1 And Kaiso in HCT116 cells.

Another area of interest that we did not fully explore here is the role of epigenetic factors in the function of Kaiso and its interaction with BRCA1. Important questions still to be answered are ‘Does SUMOylation of Kaiso affect its interaction with other members of the transcriptional complex?’ ‘Does methylation of cytosines in the eKBS effect Kaiso binding?’ As we noted in the introduction mutations in Kaiso can drive clonal haematopoiesis and CHIP phenotypes [12]. It will be interesting to address these key questions in another study where Kaiso binding to methylated promoters can be examined in detail and compared with the results shown here. Zhenilo *et al*. demonstrated that when Kaiso is deSUMOylated it is no longer able to activate expression of genes that regulate ion transport, blood pressure and immune response, though its repressor activity is not altered [19]. Kaplun *et al.* reported that when Kaiso binds to methylated DNA it recruits additional co-repressors that can affect not only chromatin structure but also gene expression [41,42]. Moreover, KO of Kaiso in renal carcinoma cells results in increased expression of pluripotency genes involved in genetic reprogramming, such as KLF−4 and Oct4, suggesting that Kaiso is normally repressing the expression of those genes. Still another study showed that the requirement for the Kaiso-binding site to be methylated for transcriptional activation or repression will vary among cell types [43]. Interestingly, the complexity of Kaiso binding to methylated versus unmethylated DNA is exhibited by a study showing Kaiso binding to the unmethylated KBS maternal allele of imprinting control region 1 (ICR1) plays an important role in the maintenance of methylated ICR1 from the paternal allele , though another group used Kaiso deletion and Kaiso mutation to show that Kaiso was not essential for methylation imprinting of H19 ICR [44]. Moreover, Starshin *et al.* showed a slight increase in overall DNA methylation when Kaiso was knocked out in Caki1 kidney carcinoma cells, but this was accompanied by hypomethylation at enhancers and gene bodies. Using Kaiso antibodies developed in the Reynolds lab, Starshin showed that Kaiso exhibits a preference for binding methylated DNA [45]. Sharshin also showed that Kaiso has a role in regulating the balance of DNA methylation at specific genomic regions. They demonstrated that several gene sets representing key biological processes were upregulated when Kaiso is knocked out in Caki1 cells: rRNA processing, ribosome biogenesis, ribonucleotide complex biogenesis and RNA processing. These terms also appeared in our analysis of Go terms associated with Kaiso binding to promoters in HCT116 cells and MCF-7 cells (electronic supplementary material, figure S3I). A few of the genes noted by Starshin *et al.*, as regulated by Kaiso in Cak1 cells also appeared in our analysis of genes with Kaiso binding to their promoters: FAM83G, TSC22D1 and PPPP1R14A. Further analysis of their raw datasets would likely reveal many more commonalities. Clearly, Kaiso is playing an important and complex role in the regulation of gene expression as well as methylation and detailed analysis; its role in the regulation of each group of genes regulated by Kaiso in different cell types will be an enormous but profitable task figure 9.

## 4. Methods

### 4.1. Cell lines

HCT116 human colon cancer cells were purchased form ATCC and were cultured in McCoY5a media with 10% FBS. The HCT116-LZRS-FLAG-Kaiso cell line was previously generated in our laboratory and cultured in McCoY5a media with 10% FBS and 400 ng ml^−1^ G418. The MCF-7 I P pol line was developed in the Reynolds lab whereby DNA damage is temporarily induced in response to treatment with 4-hydroxy tamoxifen.

### 4.2. Reversible crosslinking immuno-precipitation for mass spectrometry

To identify Kaiso binding proteins, ReCLIP was performed as previously described^18^ with modifications. ReCLIP employs cell-permeable, thiol-cleavable crosslinkers and ‘in cell’ crosslinking to covalently stabilize (‘capture’) endogenous protein–protein interactions as they occur inside the cell—prior to cell lysis and processing. Interactions covalently captured *in situ* are robustly retained through vigorous/harsh washing steps until the end of the procedure, at which time they are gently recovered from the precipitation matrix by addition of the reducing agent DTT. Crosslinked nuclear extracts were used for immunoprecipitation. HCT116 cell nuclei were prepared before crosslinking. HCT116 cells were collected and washed once with phosphate buffered saline (PBS). Cells were treated with hypotonic buffer (10 mM HEPES, pH 7.9, 10 mM KCl, 1.5 mM MgCl2, with protease inhibitor cocktail and PhosSTOP phosphatase inhibitor cocktail) for 15 min on ice. Then IGEPAL-CA630 was added to give a percentage of 0.6%, and the sample was vortexed for 10 s. The homogenates were centrifuged for 2 min at 11 000*g* at 4°C, cell nuclei were resuspended in PBS. Nuclear proteins were crosslinked with 0.5 mM dithiobis-succinimidyl propionate(DSP, Thermo Scientific cat. no. 22585) and 0.5 mM dithio-bismaleimidoethane (DTM, Thermo Scientific cat. no. 22335) at RT for 30 min then centrifuged for 2 min at 11 000*g* to remove crosslink reagent. Nuclei were washed once with PBS then high salt nuclear extraction buffer (20 mM HEPES, pH 7.9, 1.5 mM MgCl2, 0.42 M NaCl, 0.2 mM EDTA, 25% Glycerol, with protease inhibitor cocktail and PhosSTOP phosphatase inhibitor cocktail) was added, and the mixture was incubated on ice for 20 min, then sonicated for 90 s. The next step was to centrifuge for 10 min at 11 000*g* at 4°C to collect nuclear extracts. Before doing immunoprecipitation, nuclear extracts were diluted 2.8 fold with nuclear extract dilution buffer (10 mM Tris.Cl, pH 7.5, 0.05% NP-40, 0.5 mM EDTA, with protease inhibitor cocktail and PhosSTOP phosphatase inhibitor cocktail). Seven mg of lysates and 60 μg of our Kaiso monoclonal antibody clone 6F, clone 12G or mouse normal IgG were used for each immunoprecipitation. Lysates were precleared with Protein G Dynabeads (Invitrogen). Antibodies were diluted with PBS with 0.05% Tween 20, Protein G Dynabeads were then added and incubated for 10 min. Lysates were added in the tube with antibody bound beads and were rotated for 2.5 h at 4°C. The beads-Ab–Ag complex was washed three times with Co-IP washing buffer (10 mM Tris.Cl, pH 7.5, 0.05% NP-40, 0.5 mM EDTA, 150 mM NaCl, with protease inhibitor cocktail and PhosSTOP phosphatase inhibitor cocktail). Kaiso binding proteins were eluted with 50 μl RIPA buffer (0.15 M NaCl, 50 mM Tris-HCl (pH 7.4), 1% NP-40, 0.1% SDS, 1% sodium deoxycholate and 5 mM EDTA) with 50 mM DTT. A small amount of elute was taken for western, the rest of the elute was used for mass spectrometry analysis. After elution, beads were boiled in 1× NuPAGE LDS-sample buffer with reducing reagent (Invitrogen) and subjected to electrophoresis and western blot to check the efficiency of ReCLIP. Eluted samples were submitted to the Vanderbilt Mass Spectrometry Core for analysis.

### 4.3. Mass spectrometry

Protein samples were brought to 5% SDS, were reduced with 35 mM DTT and alkylated with 70 mM iodoacetamide. Samples were then prepared for S-trap (ProtiFi) digestion similar to methods described previously [46]. Aqueous phosphoric acid was added to each sample for a final concentration of 1.2%, which was followed by S-trap binding buffer (90% methanol in 100 mM TEAB) at 6 times the sample volume. Samples were loaded on S-Trap micro columns, washed with S-trap binding buffer and proteins were digested with 1 ug trypsin (Promega) for 1 h at 47°C. Peptides were serially eluted with 50 mM ammonium bicarbonate, 0.2% formic acid and 0.2% formic acid in 50% acetonitrile. Peptides were dried and resuspended in 0.2% formic acid for analysis by LC-coupled tandem mass spectrometry (LC-MS/MS). Peptides were loaded onto an analytical column packed with 3 mm of C18 reverse phase material (Jupiter, Phenomenex) using a Dionex Ultimate 3000 nanoLC and autosampler and were gradient-eluted at a flow rate of 350 nl min^−1^ for a 90 min gradient. Mobile phase solvents consisted of 0.1% formic acid, 99.9% water for solvent A and 0.1% formic acid, 99.9% acetonitrile for solvent B. The LC gradient was as follows: 1−73 min, 2−40% B; 73−78 min, 40−90% B; 78−79 min, 90% B; 79−80 min, 90−2% B; 80−90 min, 2% B. Peptides were analysed using a data-dependent method on a Q Exactive Plus mass spectrometer (Thermo Scientific), with a nanoelectrospray ionization source. The instrument method consisted of MS1 (AGC target value of 3e6), followed by 15 MS/MS scans (AGC target of 1e5). Dynamic exclusion was set to 10 s, and higher-energy collisional dissociation (HCD) collision energy was 28 nce. Tandem mass spectra were searched with Sequest (Thermo Fisher Scientific) against a human protein database created from the UniprotKB database (https://www.uniprot.org/). Variable modification of +15.9949 on Met (oxidation) and +57.0214 on Cys (carbamidomethylation) were included for database searching. Search results were assembled with Scaffold 5 (Proteome Software).

### 4.4. ChIP-seq analysis

ChIPmentation was performed as previously described [47,48] with some modifications. HCT116 wild-type cells and HCT116-LZRS-Flag-Kaiso cells were collected by trypsinization and resuspended in PBS. To perform protein:protein crosslinking, disuccinimidyl glutarate was added to the cell suspension at final concentration of 2 mM and incubated for 30 min at room temperature. Then fresh formaldehyde was added to the cell suspension at final concentration of 1% and incubated for 10 min at room temperature. Glycine was added to final concentration of 0.125 M to quench the reaction. Cells were resuspended at 1 × 10^7^ ml^−1^ in a sonication buffer (10 mM Tris-HCl pH 8.0, 2 mM EDTA pH 8.0, 1% SDS, with protease inhibitor cocktail (Roche) and PhosSTOP phosphatase inhibitor cocktail (Roche) and sonicated for 25−30 s with Diagenode One sonicator (Diagenode) in a 50 μl Bioruptor One to achieve DNA fragments in the size range of 200−700 bp.

After sonication, the lysate was adjusted to RIPA buffer conditions (10 mM Tris-HCl pH 8.0, 1 mM EDTA pH 8.0, 140 mM NaCl, 1% Triton X-100, 0.1% SDS, 0.1% sodium deoxycholate, with protease inhibitor cocktail and PhosSTOP phosphatase inhibitor cocktail). For each immunoprecipitation, lysate from 2 × 10^6^ cells and 6 μg of antibody were used. Rabbit anti-BRCA1 (Bethyl Laboratories) were used for chromatin immunoprecipitation. Forty μl Dynabeads^TM^ Protein G (Invitrogen) or Dynabeads^TM^ Protein A (Invitrogen) were used in each immunoprecipitation. After immunoprecipitation, beads were washed twice with RIPA low-salt buffer, twice with RIPA high-salt buffer, twice RIPA lithium-chloride buffer and once with 10 mM Tris-HCl buffer (pH 8.0). Illumina sequencing adapters were added on bead-bound DNA fragments via tagmetation using Illumina Tagment DNA TDE1 Enzyme and Buffer Kits (Illumina). Then bead-bound DNA fragments were extracted by reversing the crosslink and proteinase K digestion. DNA fragments were purified with AMPure XP beads (Beckman Coulter). Quantitative polymerase chain reaction (qPCR ) was performed first with KAPA HiFi HotStart Ready Mix and a pair of Nextera custom primers to determine the optimal polymerase chain reaction (PCR) cycles for the DNA library preparation for each immunoprecipitation. The final enriched DNA library for each immunoprecipitation was created by PCR with KAPA HiFi HotStart Ready Mix and Nextera custom primers using the optimal PCR cycles determined by qPCR. The Enriched DNA library was purified with AMPure XP beads, and size-selection was performed by controlling the concentration of ethanol in the AMPure XP beads and DNA mixture. Acquired DNA libraries were sequenced by the Illumina NovaSeq 6000 platform at Vanderbilt Technologies for Advanced Genomics core laboratory. Sequencing reads were aligned to the human reference genome GRCh38 using Bowtie2 [49]. Peaks for each sample were called by MACS2 with a q value cut-off of 0.01 and the corresponding input as the control [21,50,51]. Peaks were annotated using Homer (http://homer.ucsd.edu/homer/) and assigned to their closest genes. Enriched motifs were identified by the Homer command findMotifsGenome with the default region size and the motif length (size 200 and length 8, 10 and 12). Heatmaps and profile plots of sequencing reads across the ChIP-seq peak centres were generated using deepTools [52]. For the ZBTB33/Kaiso ChIP analysis from HCT116 cells, the data were downloaded from ENCODE and IDR threshold peaks were used. Homer was used to annotate peaks and assign peaks to genes. The ENCODE links are as follows:HCT116, POLR2A: https://www.encodeproject.org/experiments/ENCSROOOEUU/; HCT116, ZBTB33: https://www.encodeproject.org/experiments/ENCSR000BNY/; MCF7, BRCA2: https://www.encodeproject.org/experiments/ENCSR1190FH/; MCF7, POLR2A: https://www.encodeproject.org/experiments/ENCSR000DMT; MCF7, ZBTB33: https://www.encodeproject.org/experiments/ENCSR231YFE/.

### 4.5. Immunoblotting

Whole cell lysates, nuclear extracts and IP were boiled with NuPAGE lithium dodecyl sulfate (LDS)-sample buffer and reducing reagent (Invitrogen). Proteins were separated on 4−20% Precast Midi Protein Gel (BioRad) and transferred using Trans-Blot Turbo RTA Transfer Kit (Nitrocellulose, BioRad). Blots were blocked with 5% non-fat milk in TBS-T buffer for 1 h at room temperature followed by overnight incubation with primary antibody (diluted with 5% non-fat milk) at 4°C. After washing four times with Tris-buffered saline with Tween20 (TBS-T) buffer, blots were incubated with anti-rabbit HRP conjugate (Promega, cat. no. W401B, 1 : 20 000 dilution with 5% non-fat milk). After washing five times with TBS-T buffer, blots were incubated SuperSignal West Pico PLUS Chemiluminescent Substrate (Thermo Scientific, cat. no. 34580). Enhanced chemiluminescence (ECL) signals were captured with HyBlot CL autoradiography film (Thomas Scientific, cat. no. 1141 J51). Films were scanned, and signals were quantified with Image Studio Lite v. 5.2 (LI-COR).

### 4.6. Immunofluorescence staining

Immunofluorescence staining was performed to check if the FLAG-tagged Kaiso localizes at the same subcellular structures with the wild type of Kaiso. HCT116-LZRS-FLAG-Kaiso cells and wild-type HCT116 cells were seeded on coverslips coated with human fibronectin 10 μg ml^−1^ (Invitrogen), grown for 24−48 h. Cells were fixed with 4% paraformaldehyde in PBS for 30 min, permeabilized in 0.4% Triton X-100 in PBS for 10 min, washed twice with PBS and blocked in 1% BSA in PBS. The cells were then sequentially incubated with the primary antibody for 2 h. Washed 4 times with PBS, then incubated with the secondary antibodies for 1 h. To stain FLAG-tagged Kaiso, mouse anti-FLAG M2 (Sigma-Aldrich) and goat anti-mouse IgG- Alexa Fluor 568 (Molecular Probes) were used. To stain wild-type Kaiso, rabbit anti-Kaiso (Cell Signaling Technology) and goat anti-rabbit IgG Alexa Fluor 488 (Molecular Probes) were used. DAPI was used to stain cell nuclei. All fluorescent images were acquired on a Leica DMi8 microscope equipped with Leica LAS X software.

### 4.7. Proximal ligation assays

For proximal ligation, assays on parental HCT116 cells and p120 KO HCT116 cells were plated (30 K cells per well in an 8-well chamber slide) and allowed to grow for 24 h that brought the cells to a sub-confluent density (<60% confluent). Cells were treated with doxycycline (4OHT) for 24 h, fixed, permeabilized, blocked and then processed for proximal ligation assay (PLA). The Millipore sigma PLA Duokink fluorescence protocol was utilized. The 6F8 Kaiso antibody was used and a control was without antibody. To ensure the assay was working, a p120 monoclonal antibody and a p120 poly-clonal antibody were tested in parental HCT116 cells and in p120 KO HCt116 cells. The p120 KO cells did not exhibit any staining. The ZBTB33 (Kaiso) antibody (6F8 polyclonal antibody; 2 μg ml^−1^) was tested, and we observed nuclear and cytoplasmic staining in HCT116 cells but only cytoplasmic staining in p120 KO cells. The BRCA1 antibody was a rabbit polyclonal used at a concentration of 2 μg/ml as the rabbit Kaiso and p120 polyclonal antibodies. Monolink staining was performed with BRCA1 (Monolink) and Kaiso polyclonal antibodies and Duolink staining was performed for BRCA1 and Kaiso (6F antibody). There were four wells for Monolink: one for Kaiso antibody, one for BRCA1 antibody, one for 24 h post 4OHT treatment (4OHT is dissolved in ethanol and stored at −80C, then used at a concentration of 2μM—this treatment drives Kaiso to the nucleus) for Kaiso antibody and another for BRCA1 antibody 24 h post 4OHT. The second plate contained both Kaiso and BRCA1 antibody at the zero time point and after 24 h post 4OHT. Hoechst staining was used to localize nuclei.

### 4.8. Luciferase promoter reporter assays

These experiments were performed to analyse Kaiso and BRCA1 cooperativity towards transcriptional transactivation of different cell cycle and translation machinery. The genes were selected from the identified common cis-elements in promoters of protein synthesis and cell cycle genes [24]. Genes were analysed on the ChIPseq data base (UCSC genome browser), and the genomic coordinates were selected, after visualizing the Kaiso and BRCA1 occupancy, and putative Kaiso-binding site within the selected region. The selected promoter regions of the selected genes were amplified using specific primers (Integrated DNA Technologies) consisting of appropriate unique restriction sites compatible with PGL3 basic vector (Promega PGL3 system). HCT116 genomic DNA was used to PCR amplify, and after digestion and gel purification was cloned to PGL3 basic vector. The sequences were verified by DNA sequencing (table 1).

**Table 1.** Primers used to develop luciferase promoters for genes analyzed.

gene	amplicon; cloning site	chromosomal location
CCNC promoter	1001 bp; (Xho1/BglII)	100016246−100017246)
100017246) forward primer: 5’-GCG CCT CGA GGA GGG CGG ATC ACG AGG TCA G−3’		
reverse primer: 5’-GCG CAG ATC TGC GGT CTC CCG TGA GGA CCC C−3’		
CDK8 promoter	1217 bp; Xho1/BglII)	(26827784-26829000)
forward primer: 5'- GCG CCT CGA GGT GGC ACA AAA TGT GTT TCT AA T GCA AAC G −3'		
reverse primer: 5'- GCG CAA GCT TGG CAC CCC GGC GGC CCG CTC TC −3'		
CDC5L promoter	1039 (Xho1/HindIII)	(44354800-44355838)
forward primer: 5'- GCG CCT CGA GGG CGA TGA A TT AAA AAC TCT GAC −3’		
reverse primer: 5'- GCG CAG ATC TTA ACA AGT GAG CTT GGA GCC GAG−3’		
RPS9 promoter	1001 bp; (Nhe1/BglII)	(54704237-54705237)
forward primer: 5'- GCG CGC TAG CGG GGC CAC GGC GGG AGG CTC TCT C−3’		
reverse primer: 5'- GCG CAG A TC TGG ACT AGA TAG AGT ACA TGG GCA CCT TC −3’		
RPS11 promoter	1116 bp; (Nhe/BglII)	(49998994-50000109)
forward primer: 5'- GCG CGC TAG CCA CCA TGC CCG ACT AAT TTT G −3’		
reverse primer: 5'- GCG CAG ATC TGC CCC CAG GAA TT A GGA AGC −3‘		
RPS19 promoter	1703 bp (Xho1/BglII)	
forward primer: 5'- GCG CCT CGA GTT ATG CT A GAA GCA TGG TTG −3’		
reverse primer: 5'- GCG CAG ATC TAA CTC CTG CTG GTT CAC GTC −3’		
HNRNPK-P1 promoter	190 bp (Xho1/BglII)	(86595483-8659672)
forward primer: 5'- CGC GCT CGA GCT CCA ACC CCA AAG GAC TC −3’		
reverse primer: 5'- CGC GAA GCT TAT AAT GGC GTC TGC AGT GCT −3		
Kaiso primer:		
forward primer: 5’-AGT TGT TCT ATC TCG CGA GAG GTT CGC CCC C−3’		
reverse primer: 5’-GGG GGC GAA CCT CTC GCG AGA TAG AAC AAC T−3’		
GFP-Kaiso primer:		
forward primer: 5’-GAG ACT CGA GAT GGT GAG CAA GGG CAG GAG−3’		
reverse primer: 5’-GAA ATC AGT TTT CTA CTC TCC ATC TTG TAC AGC TCG TCC ATG CC−3’		
GFP-Kaiso primer: second set		
forward primer: 5’-GGC ATG GAC GAG CTG TAC AAG ATG GAG AGT AGA AAA CTG ATT TC−3’		
GFP-Kaiso primer: third set		
forward primer: 5’-GCT CCA ACA CAT CTG ACT CC−3’		
reverse primer: 5’-GGG AGT CAG ATG TGT TGG AG−3’		
DN Kaiso		
forward primer: 5’-ATG GAG AGT AGA AAA CTG ATT TCT GCT ACA GAC GTC CCA TTG TCA CAG GTT AAA AGC−3’		
reverse primer: 5’-GAG AGG ATC CCT AGT AAG ACA CTG GTA TTT TAA ATT C−3’		
BRCA1 primer:		
forward primer: 5’-GGC AAC GAA ACT GGA CTC ATT ACT C−3’		
reverse primer: 5’-TAG ACA GAC ACT CGG TAG CAA CGG 3’		

BRCA1 was cloned using an entry vector obtained from the Harvard Plasmid Repository via the Gateway cloning system into the pDonr LZRS-Neo plasmid(Gateway-14772 bp vector). The construct was sequence-verified. GFP-tagged Kaiso was cloned into the pcDNA3.1 vector. The green fluorescent protein (GFP)-tagged Kaiso construct was cloned into the pcDNA3.1 vector, and 12−117aa (BTB Poz domain) were deleted from the GFP-kaiso pcDNA 3.1, to generate GFP-DNKaiso. Construct DNA was transfected into HEK293 cells at the following concentrations: Initially, promoter DNA was titrated for optimal luciferase expression (50, 100, 150, 200, 250 and 300 ng), and finally 100 ng of the promoter DNA was selected as optimal. This was transfected into the HEK293 cells along with empty pGL3 basic as control, and phRL CMV (5 ng per well) was used per transfection in a 12-well format. The transfections were done in triplicate. A dual luciferase kit (Promega) was used to measure the luciferase activity as per the manufacturer’s instruction. A Renilla control was used to normalize for transfection efficiency. The Renilla luciferase reading (averaged from four wells) was used as a denominator to normalize, the pGL3 promoters (cloned) and other experimental wells (combine different plasmids) in (firefly luciferase) readings. Relative fold change was calculated; as per pGL3 basic plasmid vector (empty vector).

To investigate the cooperative role of Kaiso and BRCA1 in the transcriptional activation of genes involved in cell cycle regulation and the translational machinery, the promoter regions of selected genes (as previously described) were analysed. The genes were initially selected based on findings from *Acta Biochimica Polonica* [24]. Occupancy of Kaiso and BRCA1 at the corresponding promoter regions was confirmed using ChIP-seq data from the hg19 assembly available on the UCSC Genome Browser. Promoter regions of the selected genes were PCR-amplified from HCT116 genomic DNA using specific primers containing restriction enzyme sites compatible with the pGL3-Basic vector (Promega). The amplified products were digested, gel-purified and subsequently cloned into the pGL3-Basic vector. All cloned sequences were verified by Sanger sequencing. Luciferase activity was measured as described above. Promoter DNA was initially titrated to determine optimal expression levels. For each transfection (performed in 12-well plates and in triplicate), 100 ng of promoter construct DNA, 5 ng of phRL-CMV (as internal control) and pGL3-Basic (as negative control) were used. The pBluescript DNA was used to compensate the DNA quantity (all the wells had equivalent total DNA). Statistical analysis was performed using GraphPad Prism using one-way analysis of variance with Bonferroni’s multiple comparison test; *p* < 0.05 was considered significant.

## Data Availability

The raw data from the BRCA1 ChIPseq experiments in HCT116 cells has been submitted to the GEO database and the code is GSE294265. Supplementary material is available online [53].
